# Pharmaco-nutraceutical improvement of the response to obeticholic acid with omega-3 polyunsaturated fatty acids

**DOI:** 10.1042/BCJ20253113

**Published:** 2025-08-18

**Authors:** Audrey-Anne Lavoie, Ariane Thérien, Anisia Silva, Emanuel Paré, Anna Ciešlak, William Gagnon, Clémence Desjardins, Mélanie Verreault, Jocelyn Trottier, Marie-Claude Vohl, Jean-Philippe Drouin-Chartier, Jacques Corbeil, Alexandre Caron, Olivier Barbier

**Affiliations:** 1 Laboratory of Molecular Pharmacology, Endocrinology-Nephrology, CHU de Québec Research Center, Québec, QC G1V 4G2, Canada; 2 Faculty of Pharmacy, Université Laval, Québec, QC G1V 0A6, Canada; 3 Centre Nutrition, santé et société (NUTRISS), Institute of Nutrition and Functional Foods (INAF), Université Laval, Québec, QC G1V 0A6, Canada; 4 Quebec Heart and Lung Institute, Quebec City, QC G1V 4G5, Canada; 5 CHU de Québec Research Center, Université Laval, Québec, QC G1V 4G5, Canada; 6 Faculty of Medicine, Québec, QC G1V 0A6, Université Laval, Canada

**Keywords:** autoimmune liver diseases, bile acid detoxification, drug response improvement, inflammation and fibrosis resolution, n-3 polyunsaturated fatty acids

## Abstract

Obeticholic acid (OCA) is the second-line therapy for primary biliary cholangitis. While efficient in promoting bile acid (BA) detoxification and limiting liver fibrosis, its clinical use is restricted by severe dose-dependent side effects. We tested the hypothesis that adding n-3 polyunsaturated fatty acids (PUFAs), eicosapentaenoic (EPA) and docosahexaenoic (DHA) acids to OCA may improve the therapeutic effect of the low drug dosage. Several liver cell lines were exposed to vehicle, low or high OCA dose (1–20 μM) in the presence or absence of EPA/DHA for 24 h. To induce ER stress, apoptosis, and fibrosis, HepG2 cells were exposed to a 400 μM BA mixture or to 2 ng/ml transforming growth factor-β (TGF-β). For inflammation analyses, THP-1 cells were activated with 100 ng/ml lipopolysaccharides (LPS). The impact of OCA+EPA/DHA was assessed using transcriptomic (qRT-PCR), proteomic (ELISA, caspase-3), and metabolomic (LC-MS/MS) approaches. The addition of EPA/DHA reinforced the ability of low OCA dose to down-regulate the expression of genes involved in BA synthesis (CYP7A1 and CYP8B1) and uptake (NTCP) and to up-regulate the expression of *MRP2* and *3* genes. EPA/DHA also enhanced the anti-inflammatory response of the drug by reducing the expression of the LPS-induced cytokines: tumor necrosis factor α (TNFα), interleukin (IL)-6, IL-1β, and monocyte chemoattractant protein-1 in THP-1 macrophages. OCA+EPA/DHA decreased the expression of *BIP*, *CHOP*, and *COL1A1* genes and the caspase-3 activity. EPA+DHA potentiate the response to low OCA doses on BA toxicity and provide additional benefits on ER stress, apoptosis, inflammation, and fibrosis. These observations support the idea that adding n-3 PUFAs to the drug may reduce the risk of dose-related side effects in patients treated with OCA.

## Introduction

Obeticholic acid (OCA; Ocaliva®) is a 6α-ethyl semi-synthetic derivative of the bile acid (BA) chenodeoxycholic acid (CDCA) [1]. BAs are hepatic cholesterol metabolites that act as natural detergents to facilitate dietary lipids absorption in the small intestine [2,3]. They also function as endocrine, paracrine, autocrine, and intracrine hormones to regulate numerous physiological functions such as energy metabolism, inflammation, immunomodulation, and microbiome composition [3,4]. For this purpose, they activate several nuclear and membrane receptors, among which the Farnesoid X-Receptor (FXR) serves as a key metabolic regulator and integrator of glucose, lipid, and energy metabolism [3,4]. This receptor also plays a predominant role in modulating BA synthesis, transport, and metabolism [2–4]. Specifically, FXR regulates the enterohepatic circulation of BAs by controlling transcription of key regulatory genes involved in their synthesis, biliary secretion, and trans-intestinal transport [2].

Upon BA activation, FXR regulates the expression of target genes through direct and indirect mechanisms that involve the formation of an active heterodimer with its partner, Retinoic X Receptor alpha (RXRα) [2,4]. In target gene promoters, the FXR/RXRα heterodimer binds to a specific DNA sequence called FXR response element [2,4]. In hepatocytes, an important FXR-activated gene encodes for the Small Heterodimer Partner (SHP) protein. This small transcription factor forms inactive complexes with the Liver Receptor Homolog-1 (LRH-1) and inhibits the LRH-1-dependent transactivation of the cytochrome P450 (CYP)7A1 gene [2,4]. The CYP7A1 gene encodes the 7α-hydroxylase enzyme that catalyzes the rate-limiting step of BA synthesis [3]. The FXR-SHP-CYP7A1 pathway is the most efficient intracrine mechanism allowing BAs to inhibit their own production [4]. In addition to CYP7A1, FXR also down-regulates several other hepatic genes that encode BA-synthesizing enzymes such as CYP8B1 and CYP27A1, as well as the BA influx transporter Na^+^-taurocholate cotransporting peptide (NTCP) [5]. More direct regulatory events (i.e. direct binding to target gene promoters) also allow FXR to induce the expression of BA efflux transporters genes multidrug-resistance protein (MRP) 2 and 3, and BA-metabolizing enzymes such as the UDP-glucuronosyltransferase 2B4 [5,6]. These regulatory mechanisms prevent BA accumulation in liver cells, making FXR a target for drugs used to treat BA-related pathologies. Due to their detergent properties, BAs are cytotoxic at high concentrations [4,7]. Their accumulation in the cholestatic liver exacerbates hepatic damage by activating cell death pathways, fibrosis, endoplasmic reticulum (ER) stress, and inflammation [8].

Compared with CDCA, OCA has a 100-time greater affinity for FXR binding [9]. Due to its anti-cholestatic effects, Ocaliva® was FDA-approved in 2016 as a second-line therapy for patients with primary biliary cholangitis (PBC) with incomplete response to Ursodiol® (URSO, UDCA) [10] and as a first-line therapy for PBC patients intolerant to UDCA [11]. It is also under clinical investigation for primary sclerosing cholangitis (PSC) [12]. PBC and PSC are rare inflammatory and autoimmune hepatobiliary diseases [10,13,14]. In these diseases, the progressive destruction of bile ducts leads to chronic inflammation, cholestasis, and fibrosis that can evolve into cirrhosis and, ultimately, liver failure [15]. Major symptoms of PBC and PSC include pruritus, fatigue, and abdominal pain [16,17]. OCA therapy significantly improves liver enzymes [i.e. alkaline phosphatase (ALP)] and histologic disease features, such as ductular injury, fibrosis, and collagen morphometry in PBC patients [18–21]. Unfortunately, OCA presents a narrow therapeutic index and has led to accidental overdoses and frequent dose-related side effects [22]. In 2020, the FDA announced restrictions on the use of Ocaliva® in patients having PBC with advanced cirrhosis of the liver because it can cause serious harm. When inappropriately dosed, OCA was associated with an increase in pruritus, fatigue, and development of cirrhosis [11,20]. In January 2025, the European Union has revoked the conditional approval for OCA based on the recommendation of the European Medicines Agency [20,23].

Several studies have highlighted the potential benefits of omega-3 polyunsaturated fatty acids (n-3 PUFAs), namely eicosapentaenoic (EPA) and docosahexaenoic acid (DHA) in treating cholestatic diseases such as PBC and PSC: 1) n-3 PUFAs protect hepatocytes against BA toxicity [24–27]; 2) n-3 PUFAs protect against PBC/PSC symptoms and complications such as necro-inflammatory liver injury [28], hepatic fibrosis [29], and hepatocellular carcinoma [30]; and 3) n-3 PUFAs regulate BA-related genes, such as CYP7A1 [24,31] and NTCP [32]. The idea of using n-3 PUFAs to treat chronic cholestatic liver diseases has emerged after the observation that DHA significantly reduced ALP levels in PSC patients [33]. More recently, our research team has observed that combining EPA/DHA with Ursodiol® enhanced the response to low drug concentrations improving pro-inflammatory BA detoxification, apoptosis, ER stress, and inflammation *in vitro* [25]. In the present study, we report that EPA/DHA potentializes the OCA-dependent reduction in BA secretion and fibrosis, while also reducing caspase-3 activity, ER stress, and inflammation.

## Results

### N-3 polyunsaturated fatty acids improve the obeticholic acid-dependent modulation of genes controlling bile acids synthesis and metabolism

**Figure 1:**
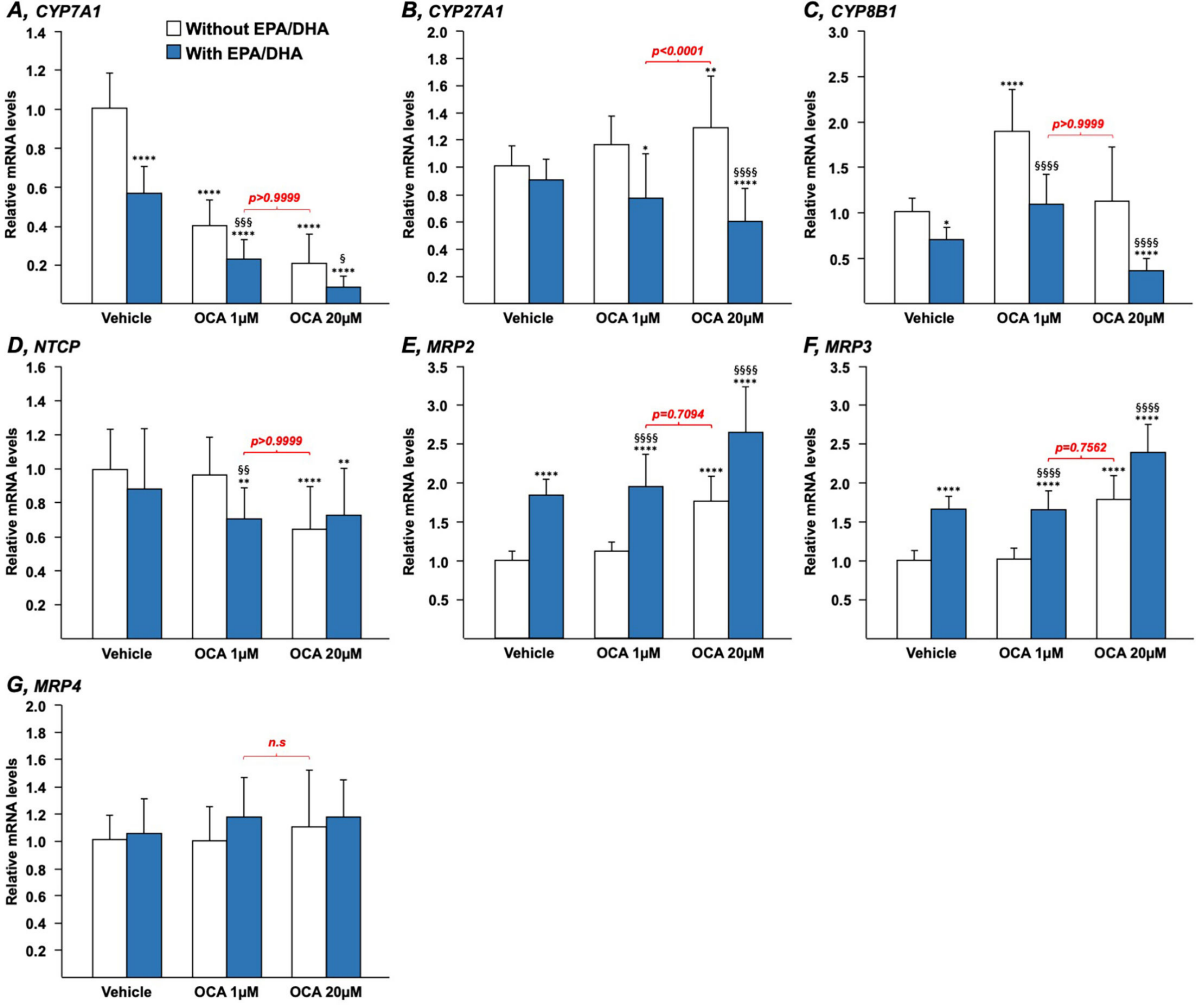
N-3 PUFAs potentiate the effects of low OCA concentrations on the expression of genes governing bile acid synthesis and transport in hepatoma HepG2 cells. Human hepatoma HepG2 cells were treated with vehicle (DMSO and ethanol), OCA (1 or 20 μM) in the absence or presence of EPA/DHA (40/40 μM) for 24 h. Total RNA was extracted. CYP7A1 (**A**), CYP27A1 (**B**), CYP8B1 (**C**), NTCP (**D**), MRP2 (**E**), MRP3 (**F**), and MRP4 (**G**) transcript levels were quantified by qRT-PCR as detailed in the Materials and Methods section, and mRNA levels were expressed relatively to control cells set at 1. Statistical significances as determined by a one-way ANOVA followed by Tukey’s multiple comparison *post-hoc* were as follows: vehicle vs. OCA-treated cells: *:*P*<0.05; **:*P*<0.01; ****:*P*<0.0001. OCA vs. OCA+EPA/DHA: §:*P*<0.05; §§:*P*<0.01; §§§:*P*<0.001; §§§§:*P*<0.0001. DHA, docosahexaenoic acid; EPA, eicosapentaenoic acid; MRP2, multidrug-resistance protein 2; NTCP, Na^+^-taurocholate cotransporting peptide; OCA, obeticholic acid.

**Figure 2:**
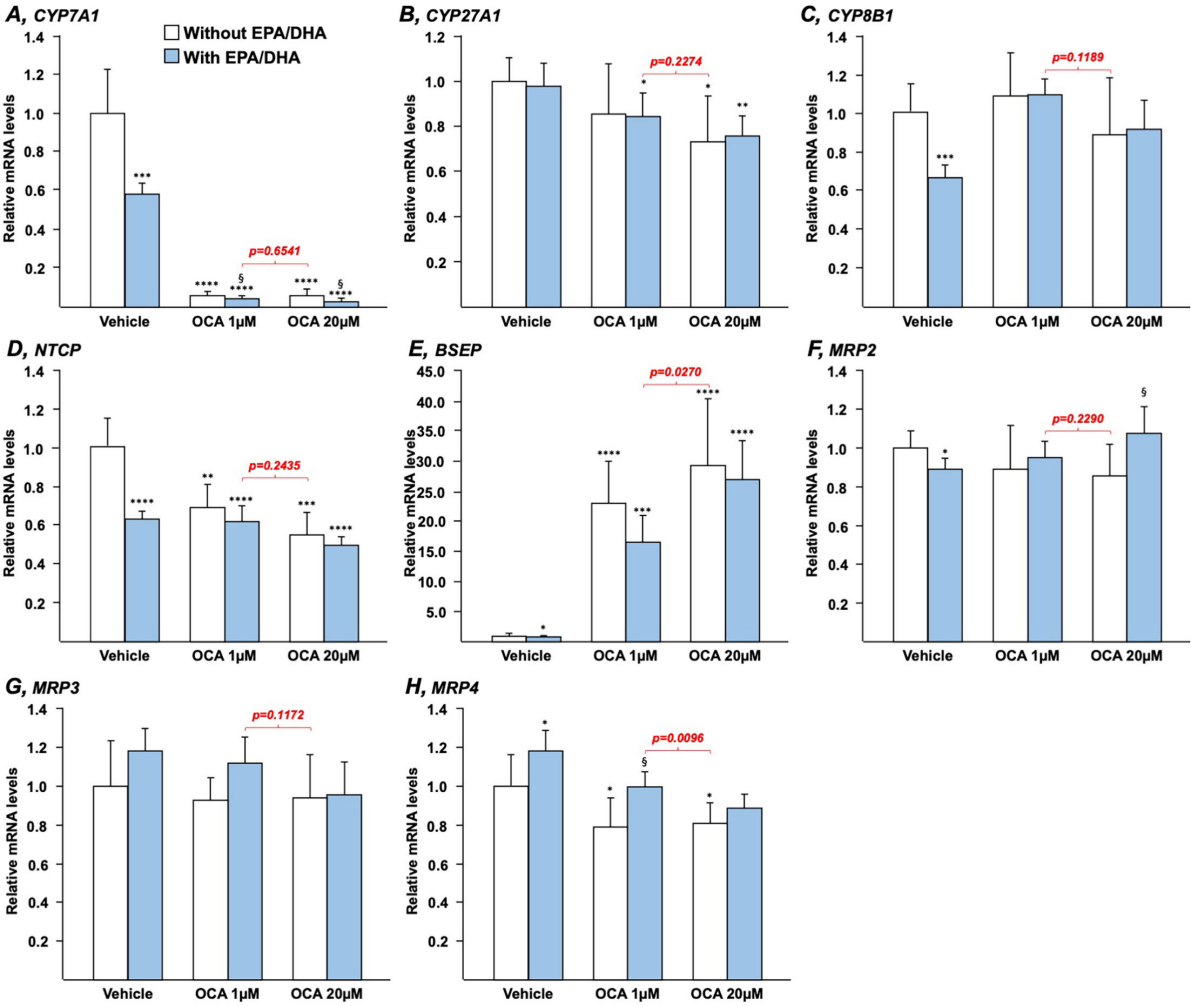
EPA and DHA modulate the ability of OCA to regulate the transcription of genes involved in bile acid homeostasis in differentiated human HepaRG hepatocytes. Differentiated human HepaRG hepatocytes were cultured in the presence of vehicle (DMSO and ethanol), EPA/DHA (50/50 µM), OCA (1 or 20 µM) or a combination of EPA/DHA (50/50 µM) and OCA (1 or 20 µM) for 24 h. Total RNA was extracted. CYP7A1 (**A**), CYP27A1 (**B**), CYP8B1 (**C**), NTCP (**D**), BSEP (**E**), MRP2 (**F**), MRP3 (**G**), and MRP4 (**H**) transcript levels were quantified by qRT-PCR as detailed in the Materials and Methods section, and mRNA levels were expressed relatively to control cells set at 1. Statistical significances as determined by a one-way ANOVA followed by Tukey’s multiple comparison *post-hoc* were as follows: vehicle vs. OCA-treated cells: *:*P*<0.05; **:*P*<0.01, ***:*P*<0.001, ****:*P*<0.0001. OCA vs. OCA+EPA/DHA: §:*P*<0.05. BSEP, bile salt export pump; DHA, docosahexaenoic acid ; EPA, eicosapentaenoic acid; MRP2, multidrug-resistance protein 2; NTCP, Na^+^-taurocholate cotransporting peptide; OCA, obeticholic acid.

**Figure 3:**
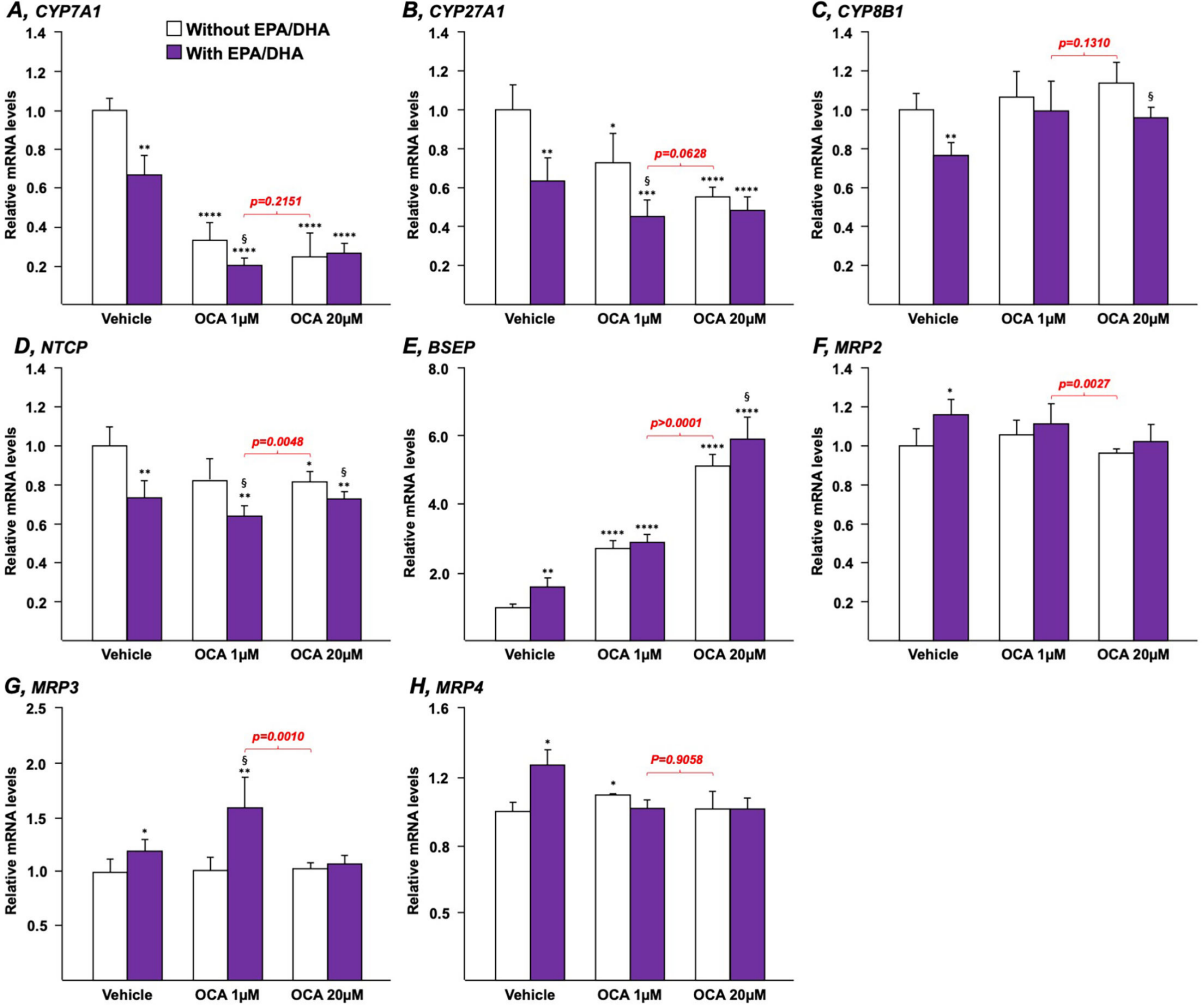
The eicosapentaenoic and docosahexaenoic acids affect the bile acid-related transcriptomic signature of OCA in primary human hepatocytes in a gene-dependent manner. Human hepatocytes in primary culture were treated with vehicle (DMSO), EPA/DHA (25/25 µM), OCA (1 or 20 µM) or a combination of EPA/DHA (25/25 µM) and OCA (1 or 20 µM) for 24 h. Total RNA was extracted. CYP7A1 (**A**), CYP27A1 (**B**), CYP8B1 (**C**), NTCP (**D**), BSEP (**E**), MRP2 (**F**), MRP3 (**G**), and MRP4 (**H**) transcript levels were quantified by qRT-PCR as detailed in the Materials and Methods section, and mRNA levels were expressed relatively to control cells set at 1. Statistical significances were as follows: vehicle vs. OCA-treated cells: *:*P*<0.05; **:*P*<0.01; ***:*P*<0.001; ****:*P*<0.0001. OCA vs. OCA+EPA/DHA: §:*P*<0.05. BSEP, bile salt export pump; DHA, docosahexaenoic acid; EPA, eicosapentaenoic acid; MRP2, multidrug-resistance protein 2; NTCP, Na^+^-taurocholate cotransporting peptide; OCA, obeticholic acid.

**Figure 4:**
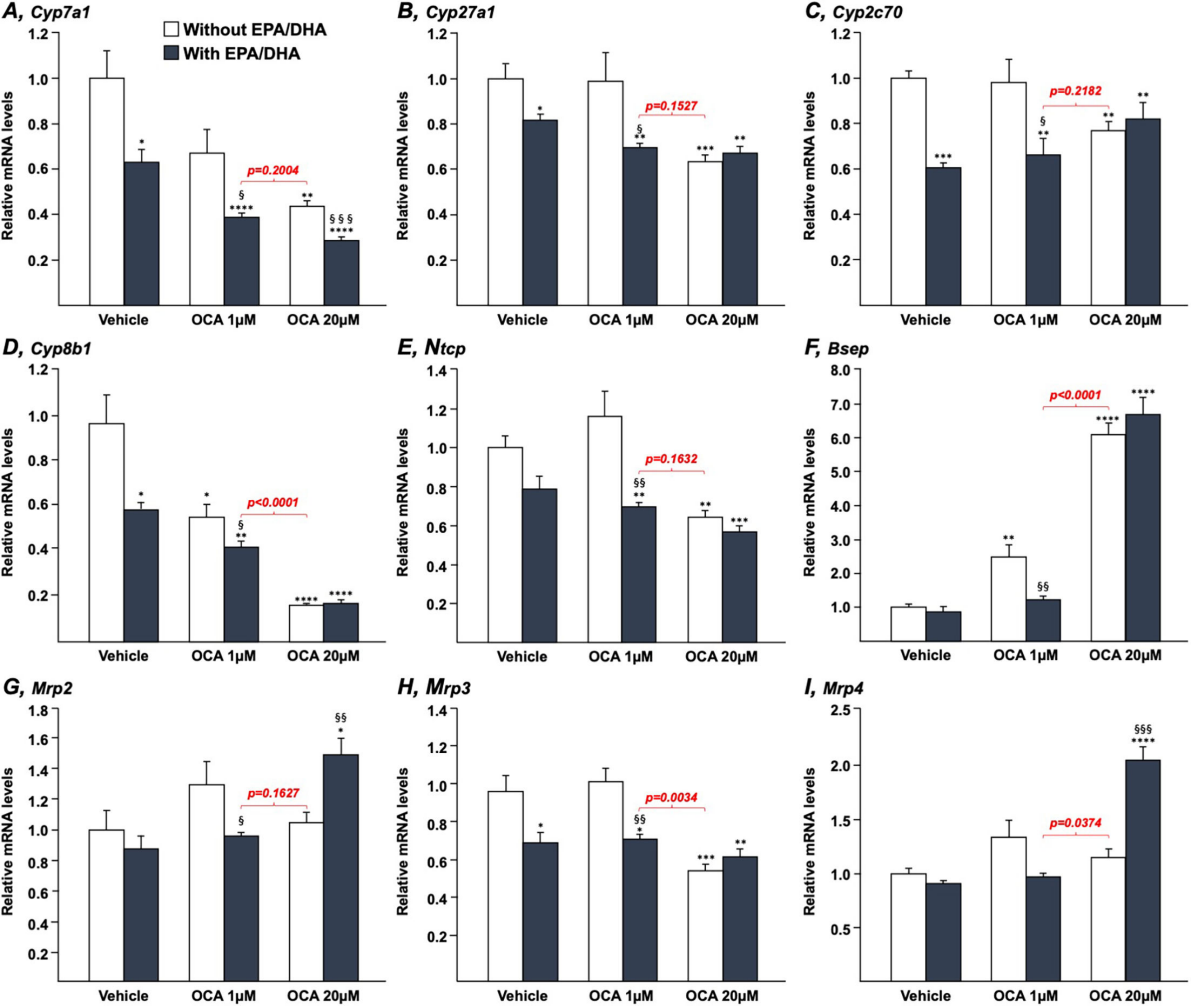
N-3 PUFAs modulate the bile acid-related transcriptomic signature of the obeticholic acid in murine hepatocytes in primary culture. Hepatocytes were purified as described in the Materials and Methods section and subsequently cultured in the presence of vehicle (DMSO and Ethanol), OCA (1 or 20 μM) in the absence or presence of EPA/DHA (50/50 μM) for 6 h. Total RNA was extracted. Cyp7a1 (**A**), Cyp27a1 (**B**), Cyp2c70 (**C**), Cyp8b1 (**D**), Ntcp (**E**), Bsep (**F**), Mrp2 (**G**), Mrp3 (**H**), and Mrp4 (**I**) transcript levels were quantified by qRT-PCR. MRNA levels were expressed relatively to control cells set at 1. Statistical significances were as follows: vehicle vs. OCA-treated cells: *:*P*<0.05; **:*P*<0.01; ***:*P*<0.001; ****:*P*<0.0001. OCA vs. OCA+EPA/DHA: §:*P*<0.05; §§:*P*<0.01; §§§:*P*<0.001. DHA, docosahexaenoic acid; EPA, eicosapentaenoic acid; MRP2, multidrug-resistance protein 2; NTCP, Na^+^-taurocholate cotransporting peptide; OCA, obeticholic acid.

Both OCA and n-3 PUFAs modulate the expression of key genes involved in the control of BA synthesis, transport, and/or detoxification [4,24,25,34]. To determine whether their effects could be additive or synergic, HepG2 (Figure 1) and HepaRG (Figure 2) cells, as well as human (Figure 3) or murine (Figure 4) hepatocytes in primary culture, were cultured in the presence of either OCA (1 or 20 µM), EPA/DHA (25, 40, or 50 µM each, as indicated) or combinations of OCA+EPA/DHA.

In HepG2 cells, and in the absence of n-3 PUFAs (Figure 1, white bars), OCA decreased CYP7A1 mRNA levels in a dose-dependent manner (Figure 1A). CYP27A1, NTCP, MRP2, and MRP3 transcripts were also significantly affected, but only in the presence of the highest OCA dose (Figure 1B, D, E and F), while CYP8B1 mRNAs were two times increased only with cells exposed to the low OCA dose (Figure 1C). MRP4 mRNA levels remained unchanged (Figure 1G). When added to OCA 1 μM (Figure 1, blue bars), EPA and DHA significantly increased the reduction rate of CYP7A1 mRNA levels from 60% (w/o EPA/DHA) to 78% (with EPA/DHA) (Figure 1A). N-3 PUFA addition to low and high OCA doses also resulted in significant changes in the expression of CYP27A1, MRP2, and MRP3 (Figure 1B, E and F). In the presence of OCA 20 μM, EPA/DHA further repressed CYP7A1 (Figure 1A), CYP27A1 (Figure 1B), and CYP8B1 (Figure 1C) mRNA expression.

Overall, these data indicate that n-3 PUFAs significantly alter the response to OCA on gene expression. These alterations could be negative such as with CYP27A1 (Figure 1B) and CYP8B1 (Figure 1C), or positive such as in the case of CYP7A1, MRP2, and 3 (Figure 1A, E and F). The most interesting observation issuing from these experiments concerns the lack of statistical significance (red *P*-values) observed when comparing mRNA levels in cells cultured in the presence of the 1 μM OCA+EPA/DHA combination versus those from cells exposed to 20 μM OCA alone (Figure 1). This is particularly the case for the expression of *CYP7A1* (*P*=0.9999), *MRP2* (*P*=0.7094), and *MRP3* (*P*=0.7562) (Figure 1A, E and F) genes. This last observation reveals not only that the addition of EPA/DHA modulates the response to a low OCA dose in terms of BA-related transcriptomic signature but also that such improvement leads to a similar response as the one obtained in the presence of a 20-time higher OCA dose.

Similar findings were also obtained when HepG2 cells were replaced by human HepaRG cells (Figure 2) or human hepatocytes in primary culture (Figure 3). For example, in HepaRG (Figure 2A) and primary hepatocytes (Figure 3A), adding n-3 PUFAs to OCA 1 μM caused a significant improvement of the drug-dependent reduction in CYP7A1 mRNA levels. As in HepG2 cells (Figure 1A), the combination of EPA/DHA to low OCA dose led to a similar response as in the presence of the highest OCA dose alone (Figures 2 and 3A). Beyond the gene expression of this rate-limiting enzyme of the BA synthesis pathway, other genes (*CYP8B1, NTCP, and MRP4*) were also affected in a similar manner in the three cell lines (Figures 1–3). By contrast, the PUFA-dependent activation of MRP2 and MRP3 transcription observed in HepG2 cells (Figure 1) was either absent (HepaRG) or limited (primary hepatocytes) in other cells (Figures 2 and 3). Finally, results obtained with hepatocytes (Figure 3E) and HepaRG (Figure 2E) reveal that the solid OCA-dependent up-regulation of the bile salt export pump (BSEP) expression is only minimally affected in the presence of EPA/DHA.

All together, these observations confirm that combining EPA/DHA to low OCA dose improves the drug-dependent inhibition of CYP7A1 mRNA expression in human hepatocytes.

### N-3 polyunsaturated fatty acids also improve the bile acid-related transcriptional signature of obeticholic acid in murine hepatocyte cells

Similar experiments were subsequently performed using murine hepatocytes (MH) in primary culture to ascertain that those benefits of the EPA/DHA plus OCA combinations also occur in liver cells from classical animal models (Figure 4). As expected [35], OCA alone caused a dose-dependent down-regulation of the murine Cyp7a1 and Cyp8b1 mRNA expression, while EPA/DHA also caused a 45% reduction in these transcripts (Figure 4A). When the two types of molecules were combined, these negative effects were additive in such a manner that the combination of EPA/DHA+OCA 1 μM led to a similar reduction as observed in the presence of OCA 20 μM alone (Figure 4A). Interestingly, EPA/DHA were found to be negative regulators of the *Cyp2c70* gene expression in MH (Figure 4C). While 20 μM OCA also down-regulated its expression, the same amount of the drug interfered with the EPA/DHA effects on this gene non-expressed in human cells and, at the basis of muricholic acid formation in rodents (Figure 4C) [2,36,37].

Beyond Cyp2c70 regulation, these experiments confirmed that, as in human hepatocytes, combining n-3 PUFAs to low OCA dose improves the drug’s ability to block Cyp7a1 mRNA expression.

### Eicosapentaenoic and docosahexaenoic acids improve the ability of obeticholic acid to reduce the production of chenodeoxycholic acid in HepG2 cells

**Figure 5:**
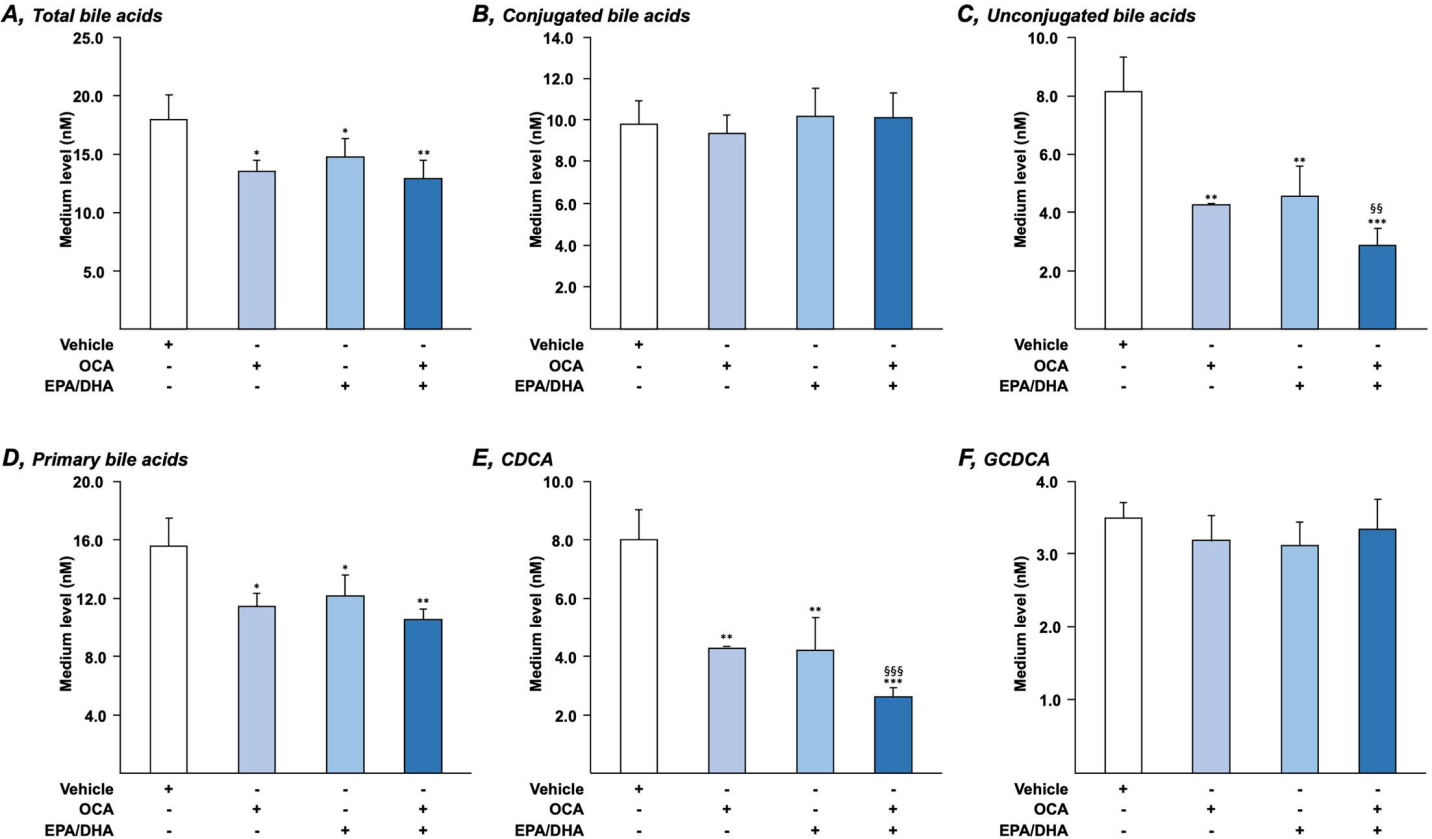
N-3 PUFAs reinforce the ability of OCA to limit bile acid secretion by HepG2 cells. Human hepatoma HepG2 cells were treated with vehicle (DMSO and Ethanol), OCA (1 μM) in the absence or presence of EPA/DHA (50/50 μM) for 36 h. Cell media were collected and profiled for the presence of 20 bile acid species using LC-MS/MS as detailed in the Materials and Methods section. Statistical significances as determined by a one-way ANOVA were as follows: vehicle vs. OCA-treated cells: *:*P*<0.05; **:*P*<0.01; ***:*P*<0.001. OCA vs. OCA+EPA/DHA: §§:*P*<0.01; §§§:*P*<0.001. CDCA: chenodeoxycholic acid; GCDCA: glycochenodeoxycholic acid.

We next investigated the impact of OCA+EPA/DHA combinations on BA secretion by HepG2 cells. As shown in Figure 5 and Supplementary Table S2, both OCA (1 μM) and EPA/DHA (50/50 μM) caused significant reductions in total (Figure 5A), unconjugated (Figure 5C), and primary (Figure 5D) BA levels. These changes mostly reflected the 50% reduction in the CDCA levels (Figure 5E). More interestingly, this reduction was further reinforced in media from cells cultured in the presence of both OCA and EPA/DHA. Other BA species, including the glyco- and tauro-conjugated forms of CDCA, remained insignificantly affected in all culture conditions (Figure 5B and F and Supplementary Table S2).

Overall, these data indicate that adding EPA/DHA to the low OCA dosage (1 μM) improves the drug-dependent inhibition of BA secretion by HepG2 cells.

### N-3 polyunsaturated fatty acids protect HepG2 cells against bile acid-induced apoptosis and ER stress in the presence of obeticholic acid

**Figure 6:**
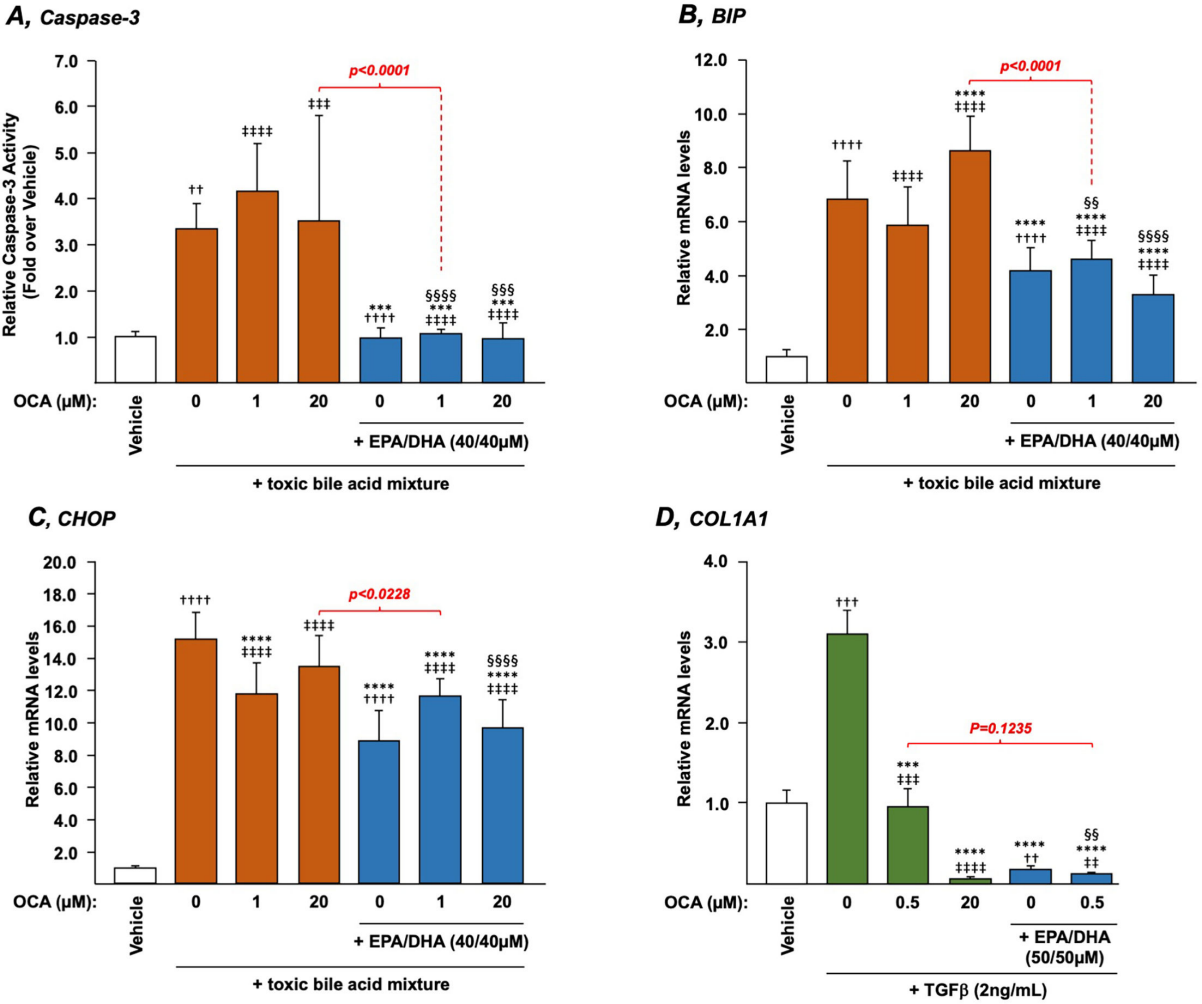
Eicosapentaenoic and docosahexaenoic acids protect liver cells against the bile acid-induced apoptosis (**A**) and ER stress (**B** and **C**), as well as against TGF-β-induced fibrosis (**D**) in the OCA-treated HepG2 (**B**) (**A**) Cells were treated with vehicle (DMSO), EPA/DHA, OCA or a combination of OCA+EPA/DHA at the indicated doses for 24 h and subsequently exposed for 2 h to the vehicle (ethanol) or a toxic BA mixture (CA, CDCA, LCA, and DCA, 100 µM each). The caspase-3 activity was then quantified as detailed in the Materials and Methods section and expressed relatively to control cells set at 1. (**B** and **C**) Cells were exposed to vehicle or the same toxic BA mixture as above for 24 h in the absence or presence of EPA/DHA, OCA (1 or 20 µM) or the OCA+EPA/DHA combinations at the indicated doses. Total RNA was extracted and analyzed for transcript levels of the ER-stress BIP (**B**) and CHOP (**C**) markers by qRT-PCR as detailed in the Materials and Methods section, and mRNA levels were expressed relatively to control cells set at 1. (**D**) Cells were treated with vehicle, EPA/DHA, OCA, or a combination of OCA+EPA/DHA at the indicated doses in the presence of TGF-β for 24 h. Total RNA was extracted and analyzed for transcript levels of the fibrosis marker COL1A1 by qRT-PCR as detailed in the Materials and Methods section, and mRNA levels were expressed relatively to control cells set at 1. Statistical significances as determined by a one-way ANOVA followed by Tukey’s multiple comparison *post-hoc* were as follows: vehicle vs. untreated cells exposed to bile acids or TGF-β: ††:*P*<0.01; †††:*P*<0.001; ††††:*P*<0.0001. Vehicle cells vs. OCA-treated cells: ‡‡:*P*<0.01; ‡‡‡:*P*<0.001; ‡‡‡‡:*P*<0.0001. BA or TGF-β-exposed cells vs. BA or TGF-β+OCA±EPA/DHA-exposed cells: ***:*P*<0.001; ****:*P*<0.0001. OCA-treated cells vs*.* OCA+EPA/DHA-treated cells: §§:*P*<0.01; §§§:*P*<0.001; §§§§:*P*<0.0001.

Because n-3 PUFAs were reported as hepatoprotective molecules against BA-induced toxicity [24,38], we next sought to determine whether EPA/DHA remained efficient in protecting HepG2 cells in the presence of OCA. For this purpose, HepG2 cells were pretreated with OCA (1 or 20 µM), EPA/DHA (40 µM each), or their combination for 24 h, and then exposed to a toxic BA mixture prior to being subjected to a measurement of caspase-3 activity. As expected [24,25], cells exposed to the BA mixture exhibited significantly higher caspase-3 activity when compared with vehicle-exposed cells (Figure 6A). In the absence of EPA/DHA, both low and high OCA doses failed to inhibit the BA-dependent caspase-3 activation. As soon as cells were pre-treated with n-3 PUFAs in the presence or absence of low and high OCA doses, BAs failed to induce caspase 3 activity (Figure 6), thereby revealing that adding n-3 PUFAs to OCA would be a means to compensate for the inability of the drug to protect liver cells against BA-induced apoptosis.

Because activation of ER-stress response has been identified as a feature of autoimmune and metabolic liver diseases [39–41], we next sought to evaluate whether OCA and/or EPA/DHA may be able to prevent the BA-induced activation of ER-stress responses [41,42]. BAs efficiently increased the relative expression of two ER-stress markers, the binding-immunoglobulin protein (BIP) and the C/EBP homologous protein (CHOP) (6.79- and 15.18-fold, respectively, *P*<0.0001) (Figure 6B and C). In the presence of 20 µM OCA, the BA-induced expression of BIP was further increased to 8.63-fold (Figure 6B). CHOP expression was repressed by low 1 µM OCA treatment (*P*<0.0001) (Figure 6C). EPA/DHA alone significantly repressed BA-induced expression of both BIP and CHOP from 6.79- to 4.14-fold and 15.18- to 8.8-fold, respectively (Figure 6B and C). When EPA/DHA were added to low and high OCA doses, CHOP mRNA levels were significantly lower than with OCA alone (Figure 6C). The combination was successful in limiting the BIP mRNA accumulation only in cells treated with 20 µM OCA (Figure 6B).

Together, these analyses revealed that the addition of EPA/DHA to OCA caused a spectacular reduction in the BA-dependent caspase 3 activation, while combining n-3 PUFAs to the drug only reduced the BA-dependent activation of the CHOP ER-stress marker.

### N-3 polyunsaturated fatty acids and obeticholic acid synergize to block the TGFβ-dependent transcription of the fibrosis marker COL1A1

OCA and n-3 PUFAs exert lowering fibrosis activities [43,44], so we next analyzed whether they can exert additive or synergistic impact on the TGFβ-dependent activation of the fibrotic marker gene collagen type I α 1 chain (COL1A1) (Figure 6D). When compared with TGFβ-activated cells, 0.5 μM OCA, EPA/DHA, and the combination caused 69.1%, 93.7%, and 96.2% reduction in COL1A1 mRNA levels, respectively (Figure 6D). Thus, indicating that adding n-3 PUFAs to low OCA dose provides a more efficient means to reduce fibrosis development in liver cells.

### The combination of EPA/DHA plus OCA suppresses the LPS-dependent induction of pro-inflammatory mediators in THP-1 macrophages

**Figure 7:**
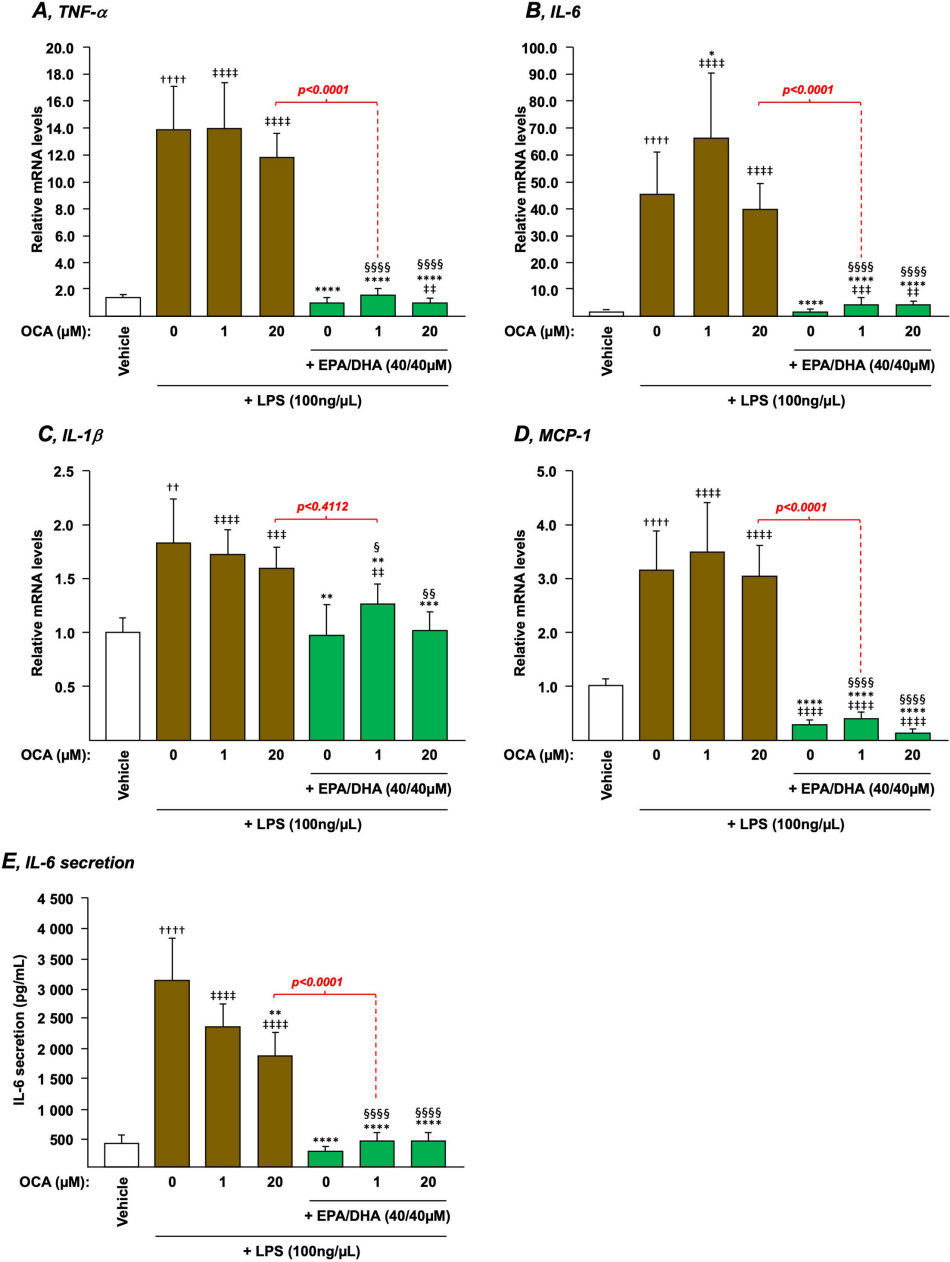
Addition of n-3 PUFAs to OCA leads to anti-inflammatory events in macrophages THP-1 monocytes were differentiated into macrophages with PMA for 72 h and then stimulated with 100 ng/μl LPS for 24 h in the presence or absence of EPA/DHA, OCA, or their combination as described in the Materials and Methods section. Total RNA was extracted. TNFα (**A**), IL-6 (**B**), IL-1β (**C**), and MCP-1 (**D**) transcript levels were quantified by qRT-PCR as detailed in the Materials and Methods section, and mRNA levels were expressed relatively to control cells (i.e. without LPS challenge) set at 1. (**E**) The level of IL-6 in culture media was measured by ELISA as described in the materials and methods section and is expressed relatively to control cells set at 1*. *Data represent the mean of three independent experiments in which each treatment was performed in triplicate. Each data point therefore corresponds to the mean of nine replicates ± SD. Statistical significances as determined by a one-way ANOVA followed by Tukey’s multiple comparison *post-hoc* were as follows: untreated differentiated THP-1 exposed to vehicle vs. untreated differentiated THP-1 exposed to LPS: ††:*P*<0.01; †††:*P*<0.001; ††††:*P*<0.0001; vehicle differentiated THP-1 vs. OCA-treated differentiated THP-1: ‡‡:*P*<0.01; ‡‡‡:*P*<0.001; ‡‡‡‡:*P*<0.0001; LPS-exposed differentiated THP-1 vs. LPS+OCA±EPA/DHA-exposed differentiated THP-1: *:*P*<0.05; **:*P*<0.01; ***:*P*<0.001; ****:*P*<0.0001; OCA-treated differentiated THP-1 vs. OCA+EPA/DHA-treated differentiated THP-1: §:*P*<0.0001; §§:*P*<0.0001; §§§§:*P*<0.0001.

We next evaluated whether OCA and/or EPA/DHA also exert anti-inflammatory effects in the classical lipopolysaccharides (LPS)-stimulated THP-1 macrophages model when used in combination. As expected [25], both mRNA levels of the inflammation markers tumor necrosis factor α (TNFα) (Figure 7A), IL-6 (Figure 7B), IL-1β (Figure 7C), and monocyte chemoattractant protein-1 (Figure 7D), as well as the secretion of IL-6 in media (Figure 7E), were markedly increased in phorbol 12-myristate 13-acetate (PMA)-differentiated THP-1 macrophages. OCA alone, either at 1 or 20 μM, remained inefficient at preventing these LPS-dependent activations (Figure 7). By contrast, all cells exposed to EPA/DHA were efficiently protected against the induction of cytokine transcription and/or secretion by LPS (Figure 7). Since the addition of EPA/DHA to 1 μM OCA provided a significantly (*P*<0.0001) higher reduction in IL-6 secretion than OCA 20 μM, one can conclude that the combination of n-3 PUFAs to low OCA dose confers a very efficient protection against LPS-induced production of pro-inflammatory mediators.

## Discussion

Using a series of complementary experimental models, the present study reveals that the addition of EPA and DHA deeply ameliorates the pharmacological impact of OCA treatment. These improvements were observed both in terms of additive impacts (such as with the reinforcement of the OCA-dependent reduction in BA secretion and fibrosis) and in terms of introduction of new benefits (as observed with the EPA/DHA-dependent reduction in caspase-3, ER-stress, and inflammation). Interestingly, the most spectacular benefits of combining n-3 PUFAs and OCA were observed in the presence of the lowest dose of the drug, thus suggesting that the addition of EPA and DHA may provide clinical benefits in terms of optimized dosage for OCA. The present study provides the very first experimental evidence that pharmaco-nutraceutical strategies combining n-3 PUFAs and low dose of OCA may be as efficient as the high drug dosage.

Despite the fact that such benefits have never been reported, the idea that pharmaco-nutraceutical combination with n-3 PUFAs could improve drug responses has already been demonstrated in the context of other experimental settings and pathological contexts, such as cancer treatment [45,46]. For example, Poulsen, R. C. and colleagues [45] reported that combining DHA to 17β-estradiol allows a lower dose of the estrogen to be used to provide similar bone-protective effects. Similarly, several studies evidenced that increasing the exposure to omega-3 PUFAs has a beneficial impact on tumor cell response to chemotherapy *in vitro* and enhances cancer chemo-sensitivity *in vivo* (reviewed in [47]). More recently, our group also reported that the addition of EPA/DHA to the first-line anti-cholestatic therapy Ursodiol® improved the drug response in a similar fashion as observed in the present investigations [25].

**Figure 8:**
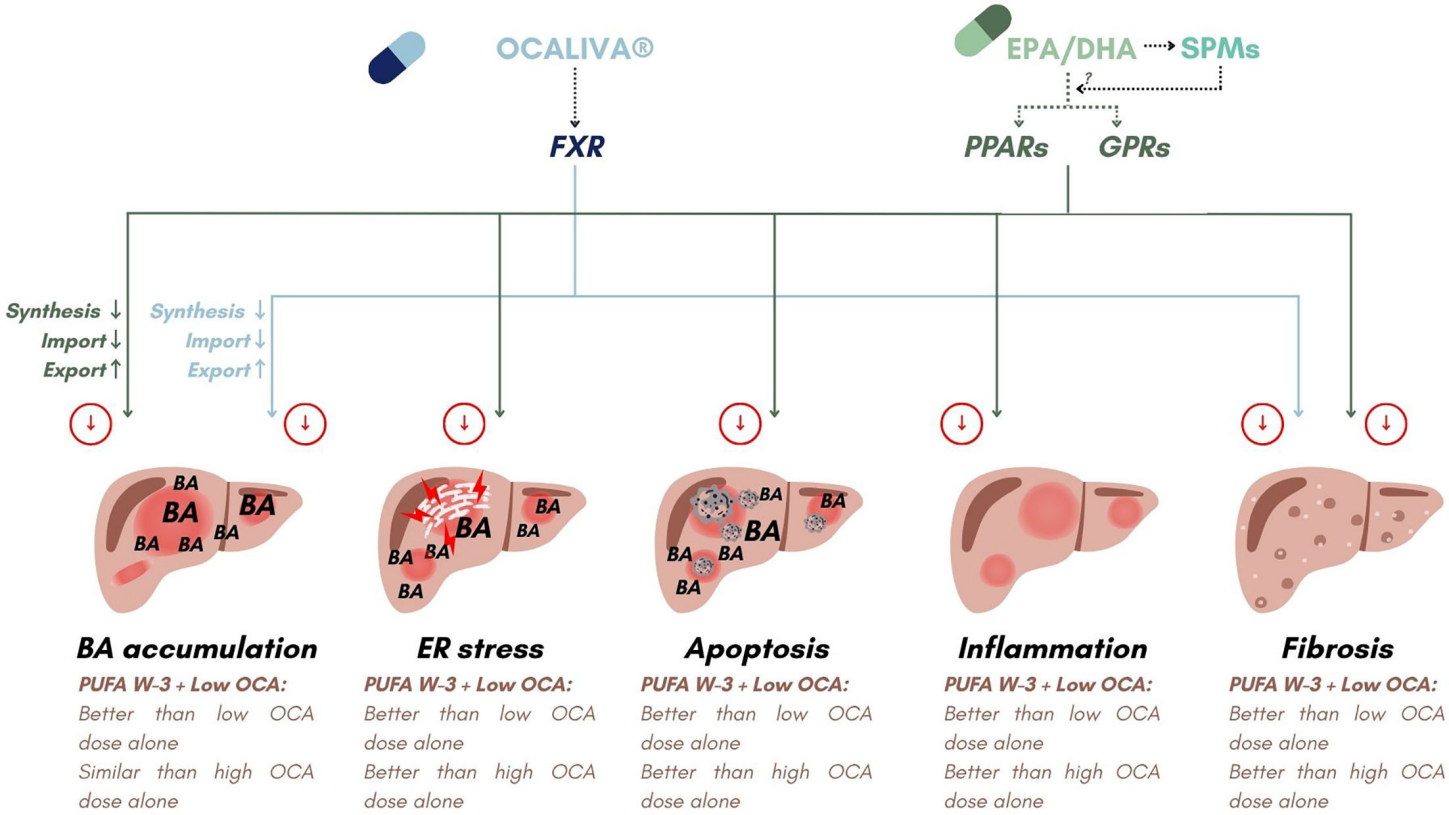
OCA and n-3 PUFAs activate different nuclear and/or membrane receptors to additively reduce the successive events involved in the pathogenesis of inflammatory and autoimmune hepatobiliary diseases. As illustrated by blue arrows, OCA controls the intracellular levels of pro-inflammatory, pro-apoptotic, and pro-fibrotic bile acids in hepatocytes through the selective activation of FXR. EPA and DHA modulate bile acid synthesis and metabolism, ER-stress, apoptosis, inflammation, and fibrosis in a direct manner through the activation of PPAR and GPCR receptors or after conversion into active SPM derivatives that also activate GPCRs. Activation of these complementary transcriptional pathways may be responsible for the additive effects observed during the present investigations. SPMs, specialized pro-resolving mediators.

With both OCA and UDCA, the addition of EPA and DHA resulted in similar improvements of the response to the drug response in terms of the BA-related transcriptome in HepG2 cells [25]. Indeed, in both cases, the drug-dependent inhibition of the rate-limiting enzyme CYP7A1 transcription was reinforced in a dose-dependent manner in the presence of PUFAs [25]. Similar effects were also observed with other genes, such as NTCP and MRPs, which are regulated by both drugs [25]. Interestingly, while the CYP27A1 gene expression was unaffected by OCA in the present study, its transcription was also additively down-regulated when UDCA was combined to EPA/DHA [25]. Taken together, these observations indicate that the benefits of n-3 PUFAs combined with anti-cholestatic drug primarily reflect the additive effects of their respective actions, rather than the reinforcement of mechanism of action of the drugs. In fact, OCA and n-3 PUFAs regulate overlapping and complementary transcriptional pathways (Figure 8). OCA is a selective FXR agonist that controls the intracellular levels of pro-inflammatory, pro-apoptotic, and pro-fibrotic BA levels in hepatocytes [34] (Figure 8). EPA and DHA act as activators of the nuclear peroxisome proliferator-activated receptors (PPAR) alpha or gamma, as well as through transmembrane specific G-protein-coupled receptors (GPCRs), such as GPCR120 [48–50] (Figure 8). These PUFAs are also converted into biologically active metabolites, such as lipoxins, resolvins, maresins, and protectins that also activate several GPCRs [50]. Interestingly, FXR, PPARs, and GPCRs are well-established controllers of BAs metabolism, inflammation, and/or fibrosis, which may allow the additive impact of the combination of OCA+EPA/DHA (Figure 8).

On the other hand, several key genes in hepatic BA uptake (NTCP) and clearance (MRP2 and MRP3) were only modulated by the supratherapeutic dose equivalent of OCA (20 µM), and the addition of EPA/DHA to the lower, clinically relevant, OCA treatment (1 µM) resulted in a significant modulation equivalent to the higher OCA dose. Considering that a reduction in BA synthesis and accumulation in liver cells remains the primary pharmacological target of Ocaliva® in the context of cholangitis treatment, these observations suggest that adding n-3 PUFAs may be an efficient way to reduce the drug dosage required to reach a maximal pharmacological impact. Another characteristic of the drug that was improved in the presence of n-3 PUFAs is its ability to prevent hepatic fibrosis as measured through the TGFβ-dependent induction of the *COL1A1* gene expression in HepG2 cells. While OCA is a well-known reducer of fibrosis and collagen fiber morphometry [51], the potential of n-3 PUFAs to control liver fibrosis is more controversial [52]. Nevertheless, in the experimental settings used in the present study, EPA/DHA were extremely efficient in limiting the TGFβ-induced *COL1A1* expression. More importantly, when added to a dose as low as 0.5 µM OCA, these fatty acids led to a complete normalization of the TGFβ-induced fibrosis markers. While limiting hepatic toxic BA accumulation is the main pharmacological role of Ocaliva®, controlling hepatic fibrosis development in PBC patients remains its principal therapeutic objective. Thus, our findings indicate that combining n-3 PUFAs to OCA may be an efficient way to reduce the drug dosage required to reach a maximal therapeutic effect.

In addition to improving the ability of the drug to reach its principal targets, EPA and DHA also provided therapeutic benefits that OCA alone failed to elicit. Indeed, OCA alone failed to protect HepG2 cells against BA-induced apoptosis and -ER stress, as well as the LPS-induced expression and production of pro-inflammatory mediators by THP-1 derived macrophages. N-3 PUFAs alone and when combined with OCA protected cells against these situations. Both the absence of response to OCA and the impact of EPA/DHA are in accordance with previous reports [25,53–55]. The development of inflammation and ER stress-induced apoptosis is a well-established characteristic of cholestatic liver diseases [25,38,39,41]. The present observations that n-3 PUFAs reduce these parameters further reinforce the potential of combining EPA and DHA to low OCA dose into pharmaco-nutraceutical anti-cholestatic strategies.

Such strategies are of obvious clinical interest in the current situation, where the use of Ocaliva® for the treatment of PBC is currently being called into question in various countries, notably in connection with the occurrence of serious dose-related side effects [1]. The administration of OCA at high doses (i.e. 25 mg or 50 mg once daily instead of the recommended 5 to 10 mg/day) results in dose-dependent hepatotoxicity, evidenced by elevations in ascites, portal hypertension, jaundice, and exacerbation of PBC (reviewed in [1]). In 2018, these hepatotoxic side effects prompted the FDA to issue a black-boxed warning stating that administration of OCA to PBC patients with decompensated cirrhosis can cause liver failure [1]. Later, the FDA removed its approbation for patients with decompensated cirrhosis (e.g. Child-Pugh class B or C) or compensated cirrhosis with evidence of portal hypertension, such as gastroesophageal varices, ascites, or ongoing thrombocytopenia [1]. More recently, in January 2025, Ocaliva® has lost its approbation for PBC treatment in the European Union [20,23]. Based on the herein reported observations, we proposed that a pharmaco-nutraceutical combination of low dose OCA+n-3 PUFAs will improve the safety profile of the drug. Because recent observations from the real world clearly establish the therapeutic advantage of the OCA for PBC patients with no or partial response to Ursodiol® [56], ensuring a safer and larger utilization of the drug may have profound impacts for the treatment of autoimmune and metabolic liver diseases. Indeed, Brookhart, M.A. and colleagues [56] reported a 63% reduced risk of hospitalization for hepatic decompensation, liver transplant, or death in OCA-treated versus non-OCA-treated individuals in real-world trial HEROES. Beyond cholangiopathies, recent investigations revealed that OCA also exhibited a similar pattern of therapeutic benefits overshadowed by significant side effects in patients suffering from nonalcoholic steatohepatitis [57]. Thus, one can envision that safer formulations of the drug may be useful in limiting the current epidemy of liver steatosis.

The present study has notable limitations, including its descriptive nature and reliance on *in vitro* models, as well as the absence of *in vivo* validation and exact mechanisms of action for the effects of n-3 PUFAs. Nevertheless, the present approach based on the use of multiple cellular models allowed to uncover a synergistic effect of the omega-3+OCA combination on BAs synthesis and related transcriptome. For most of the investigated genes, the different cell lines exhibited a very similar response to each treatment, whereas few of them were affected in a cell line-specific manner (Figures 1–4). For example, the *MRP2* and *3* genes’ expression was dose-dependently up-regulated by EPA/DHA only in HepG2 cells, while the increasing dose of n-3 PUFAs down-regulated Cyp8b1 mRNA expression in MH only. While the reasons for these differences remain to be fully elucidated, one could envision that such discrepancies could relate to the genomic, epigenetic, and phenotypic characteristics of each cell line and/or to the cell culture conditions. In any case, further *in vivo* investigations using appropriate animal models are needed to address these limits to enable the clinical use of low-dose OCA+n-3 PUFAs in patients with autoimmune and metabolic liver diseases. Given the ongoing controversy surrounding the clinical use of Ocaliva®, we sought that the promising results from the present study should be communicated in a timely manner.

## Materials and methods

### Reagents

BAs cholic acid (CA), CDCA, and deoxycholic acid (DCA) were purchased from Steraloids Inc (Newport, RI), and lithocholic acid (LCA) was purchased from Sigma-Aldrich (St-Louis, MO). OCA, EPA, and DHA were purchased from Cayman Chemical (Ann Arbor, MI). Cell culture media, FBS, penicillin/streptomycin, nonessential amino acids, and other cell culture reagents for HepG2 and THP-1 cells were purchased from Wisent (Quebec, Qc, CA).

### Cell culture

Primary human hepatocytes (HH; Sekisui Xenotech) corresponded to the CryostaX attaching pool of ten donors (five men and five women; lot #1910006) from Sekisui Xenotech (HPCH10+, Kansas City, KS) and were cultured according to the manufacturer’s instructions [58]. Donors were five women and five men; seven Caucasian, two Hispanic, and one Asian people and were aged from 7 to 69 years. The causes of death varied from anoxia (2), cerebrovascular accident (4), and head trauma (4). Hepatocytes were plated in 24-well plates (3.5 × 10^5^ cells/well) and maintained in InVitroGro CP medium for 48 h with medium change after 24 h as recommended by the supplier (Celsis-InVitro Technologies). Afterwards, cells were cultured in the presence of DMSO (vehicle, 0.1% v/v), OCA (1 or 20 µM), EPA/DHA (25/25 µM), or a combination of OCA+EPA/DHA at indicated concentrations for 24 h, before mRNA extraction (see below).

Primary MH were isolated from a 16-week-old male C57BL6J mouse. The animal was anesthetized with isoflurane, and the abdomen was opened to expose the inferior vena cava and the portal vein. The portal vein was cannulated, the inferior vena cava was cut, and the liver was then perfused with 50 ml of warm Liver Perfusion Medium (Gibco^TM^, Fisher Scientific Canada) at 8 ml/min for about 10 min using a peristaltic pump. Subsequently, the liver was perfused with 50 ml of warm Liver Digest Medium (Gibco^TM^, Fisher Scientific Canada) supplemented with 0.5% FBS (pH 7.4) at 5 ml/min for another 10 min. After the perfusion, the liver was carefully excised and placed in a 50-ml tube containing an isolation medium (IM) consisting of M199 (Wisent) supplemented with 10% FBS, 100 nM triiodothyronine (Sigma), 500 nM dexamethasone (Sigma), 1 nM insulin (Sigma), and 1% Penicillin–Streptomycin. The hepatic tissue was then scraped using forceps, filtered using a 100-μm sterile cell strainer, and centrifuged at 50* **g*** for 5 min. The supernatant was removed, and the pellet was resuspended in 10 ml of Percoll solution (Sigma) before being centrifuged again at 350 ***g*** for 5 min to separate dead cells. Hepatocytes were washed once with 40 ml of IM and centrifuged 5 min at 50 ***g***. Hepatocytes were resuspended in fresh IM, and cells were counted with trypan blue to assess viability. They were then plated on Cell+ six-well plates (Sarstedt, Montréal Qc, Canada) at 3.50 × 10^5^ cells/well. Cells were allowed to attach and recover for 4 h, and the medium was changed to a Maintenance Medium consisting of M199 supplemented with 1nM insulin and 1% Penicillin–Streptomycin. Cells were then washed twice with M199 and then treated with M199 supplemented with vehicle (DMSO); EPA/DHA (50/50 µM); OCA 1 µM; OCA 1 µM+EPA/DHA (50/50 µM) or OCA 20 µM+EPA/DHA (50/50 µM) for 6 h after which cells were collected for RNA extraction.

HepaRG cells [59] were kindly gifted by Prs. P. Gripon, C. Guguen-Guillouzo, and C. Trepo from INSERM U1052 / U991 (MTA #10,528AJJ10). HepaRG cells were cultured as previously reported [59,60] and were plated in six-well plates (800,000 cells per well) in Williams’ E medium supplemented with 2 mM Glutamax, 100 U/ml penicillin, 100 µg/ml streptomycin, 10% fetal calf serum, 5 µg/ml insulin, and 50 µM hydrocortisone hemisuccinate. Upon reaching confluence, HepaRG cells were cultured for two weeks, after which the culture media were supplemented with 1.7% DMSO and then cultured for another two weeks. After the process, the resulting differentiated hepatocytes and cholangiocyte-like cells were cultured as described [59,60]. Confluent and differentiated cells (in six-well plates) were cultured in the presence of DMSO (vehicle, 0.1% v/v), EPA/DHA (50/50 µM), OCA (1–20 µM), or a combination of OCA+EPA/DHA for 24 h.

HepG2 cells were obtained from the American Type Culture Collection (ATCC, Manassas, VA) and were cultured as described previously [25]. For RNA analyses, HepG2 cells were plated in 12-well plates (200,000 cells per well) and cultured in the presence of DMSO (vehicle, 0.1% v/v), EPA/DHA (40/40 µM), OCA (1–20 µM), or a combination of OCA+EPA/DHA for 24 h.

For apoptosis experiments, HepG2 cells (175,000 cells per well, 24-well plates) were pretreated for 24 h with vehicle (DMSO/ethanol) or EPA/DHA (40 µM each) and OCA (1–20 µM) individually or in combination. A BA mixture composed of 100 µM of CA, CDCA, LCA), and DCA was added for 2 h prior to caspase-3 activity measurement.

For ER-stress experiments, HepG2 cells were seeded in 12-well plates and treated for 24 h with the same BA mixture as above, in the absence or presence of EPA/DHA (50/50 µM), OCA (1–20 µM), or the EPA/DHA+OCA combinations.

For TGFβ-induced fibrosis, HepG2 cells were seeded in 12-well plates and treated for 24 h with 2 ng/ml TGF-β (SIGMA), in the absence or presence of EPA/DHA (50/50 µM), OCA (1–20 µM), or OCA+EPA/DHA.

For BAs formation experiments, HepG2 cells were plated in 12-well plates (200,000 cells per well) and cultured in the presence of DMSO (vehicle, 0.1% v/v), EPA/DHA (50 µM), OCA (1–20 µM) or OCA+EPA/DHA at indicated concentrations for 24 h.

THP-1 cells were obtained from the ATCC (Manassas, VA, U.S.A.). THP-1 monocytes (1,000,000 cells per well, six-well plate) were differentiated into macrophages by supplementing the culture medium with 100 nM PMA treatments for 72 h. TPH-1 differentiated macrophages were then stimulated with 100 ng/ml LPS (Sigma, St-Louis, MO) for 24 h in the presence or absence of either OCA (1–20 µM), EPA/DHA (50/50 µM), or OCA+EPA/DHA.

### RNA isolation, reverse transcription, and quantitative real-time polymerase chain reaction (qRT-PCR)

Total RNA isolation, reverse transcription (RT), and real-time PCR analyses were performed as previously described [24,25]. Briefly, RNA was isolated using the Tri-Reagent® protocol as recommended by the manufacturer (Molecular Research Center Inc., Cincinnati, OH, U.S.A.) [24,25]. cDNA was obtained by RT reactions with 1 µl of SuperScript™ IV Vilo™ master mix (Thermo Scientific, Life Technologies Division, Carlsbad, CA, U.S.A.) and 500 ng of isolated RNA in a final reaction volume of 5 µl. Gene mRNA expression was detected using Fast SYBR® Green real-time polymerase chain reaction master mix (Thermo Scientific, Life Technologies Division, Carlsbad, CA, U.S.A.) in an ABI ViiA7 system (Applied Biosystems, Foster City, CA, U.S.A.). Each reaction was performed in a final volume of 10 µl containing 5 µl of Sybr Fast® PCR mix, 1 µl of forward and reverse primers (see Supplementary Table S1), and 3 µl of diluted RT product. Quantitative real-time polymerase chain reaction (qRT-PCR) reactions were carried out at 95°C for 20 s, 95°C for 30 s, and annealing temperature for 20 s for 40 cycles. Threshold cycle (Ct) values were analyzed using the comparative Ct (ΔΔCt) method as recommended by the manufacturer (Thermo Scientific, Waltham, MA, U.S.A.). Target gene mRNA levels were obtained by normalizing to the endogenous reference Pumilio RNA-Binding Family Member 1 and expressed relatively to vehicle-treated cells set at 1.

Murine mRNA expression was determined using the Taqman^TM^ Fast Advanced MasterMix (Fisher Scientific, Applied Biosystems, Waltham, MA, U.S.A.) in an ABI ViiA7 system. qRT-PCR reactions were conducted at 95°C for 20 s, 95°C for 1 s, and 60°C for 20 s for 40 cycles. Each reaction contained a 7-μl MasterMix composed of 5 μl Taqman^TM^ Fast Advanced MasterMix, 1.5 μl SuperQ water, and 0.5 μl of primers (Fisher Scientific, Applied Biosystems, Waltham, MA, U.S.A.), with an additional 3 μl of diluted RT product. Ct values were analyzed using the ∆∆Ct method. Target gene mRNA levels of BA detoxification (Cyp7a1 [Mm00484150_m1], Cyp27a1 [Mm_00470430_m1], Cyp8b1 [Mm00501637_s1], Cyp2c70 [Mm00521058_m1], Ntcp [Mm_00441421_m1], Bsep [Mm_00445168_m1], Mrp2 [Mm00496899_m1], Mrp3 [Mm00551550_m1], and Mrp4 [Mm01226385_m1]) were obtained by normalizing to the endogenous reference 18S and expressed relatively to vehicle-treated cells set at 1.

### Caspase-3 assay

The caspase-3 activity was determined using Enzcheck® caspase-3 assay kit (Thermo Scientific, Life Technologies Division, Carlsbad, CA), as previously described [25]. Assays were performed according to the manufacturer’s instructions. Mean fluorescence was measured with an Infinite M1000 instrument (Tecan, Austria). Results are expressed as mean fluorescence normalized by sample protein concentration, as determined by BCA assay (Bio-Rad Laboratories, Hercules, CA).

### Bile acid quantification

The secretion of BAs in culture media was quantified through liquid chromatography coupled to tandem mass spectrometry (LC-MS/MS) using a Prominence ultra-high-pressure liquid chromatography (UHPLC) instrument (Shimadzu Scientific Instruments, Columbia, MD, U.S.A.), coupled with an API4000 instrument (Applied Biosystems, Concord, ON, Canada) using previously described methods [61]. Briefly, 300 μl of acidified media samples (1:1 media:formic acid 0.5 M) was diluted in 3 ml of methanol-0.1% formic acid containing 50 μl of internal standard (mix of CDCA-d4, DCA-d4, CA-d4, LCA-d4, TCA-d5, GCA-d4, and CDCA-24G-d5; C/D/N Isotopes Montréal, Canada) and then centrifuged at 5000 ***g*** for 5 min. Supernatants were evaporated under nitrogen and reconstituted in 100 μl water:methanol (50:50) prior injection to the LC-MS/MS system, as reported [61]. The same procedure was also applied to analytical standards initially diluted in blank media. Five microliters of samples or analytical standards was then injected into the chromatographic system consisting of a Prominence UHPLC instrument (Shimadzu Scientific Instruments, Columbia, MD, U.S.A.). The chromatographic separation was achieved with a C_18_ column from Agilent (150 × 2.1 mm Poroshell 120 EC-C18; 2.7 μm particles; Santa Clara, CA) at 45°C, and the following mobile phases: solvent A=ammonium acetate in water (6 mM) at pH 7.7 and solvent B=acetonitrile. Separation was performed at a flow rate of 0.35 ml/min using the following sequence: 84% A:16% B as initial conditions held for 0.5 min and increased to 27% B in 16.5 min, then a linear gradient to 31% B over the next 16 min, followed by an increase in B to 56% in 8 min. The column was then flush at 95% B over the next 12 min and back to initial conditions for 8 min. All analytes were quantified by LC-MS/MS using an API4000 instrument (Applied Biosystems, Concord, ON, Canada). The temperature was set at 500°C.

### ELISA

Interleukin (IL)-6 secretion from THP-1 cells was determined using Human IL-6 DuoSet® (R&D Systems, Minneapolis, MN, U.S.A.) and Human Tumor Necrosis Factor Alpha (TNF-α) (Invitrogen, Carlsbad, CA, U.S.A.) ELISA kits following the manufacturers’ instructions. Cytokine production was expressed relatively to vehicle-treated cells, as previously described [25].

### Statistics

Differences in cell responses between treatments were determined by one-way ANOVA followed by Tukey’s multiple comparison test *post-hoc*. *P*-values <0.05 were considered statistically significant. Statistical analyses were performed using GraphPad Prism version 7.0 (GraphPad Software, La Jolla, CA, http://www.graphpad.com). Reported *P*-values for comparisons between treatment groups are multiplicity adjusted.

## Supplementary Material

Online supplementary material 1

## Data Availability

All data will be made available upon request to the corresponding author.

## Abbreviations

ALP: alkaline phosphatase
BA: bile acid
BIP: Binding-immunoglobulin protein
BSEP: bile salt export pump
CA: cholic acid
CDCA: chenodeoxycholic acid
CHOP: C/EBP homologous protein
COL1A1: collagen type I α 1 chain
CYP: cytochrome P450
DCA: deoxycholic acid
DHA: docosahexaenoic acid
EPA: eicosapentaenoic acid
FXR: Farnesoid X-receptor
GPCRs: G-protein receptors
HH: Human hepatocytes
IL: interleukin
LCA: lithocholic acid
LC-MS/MS: Liquid chromatography coupled to tandem mass spectrometry
LPS: lipopolysaccharides
LRH-1: Liver Receptor Homolog-1
MCP-1: monocyte chemoattractant protein-1
MH: murine hepatocytes
MRP: Multidrug-Resistance Protein
NTCP: Na^+^-taurocholate cotransporting peptide
OCA: obeticholic acid - Ocaliva®
OSTβ: Organic solute transporter β
PBC: primary biliary cholangitis
PMA: Phorbol 12-myristate 13-acetate
PSC: primary sclerosing cholangitis
PUFAs: Omega-3 polyunsaturated fatty acids
RXRα: Retinoic X Receptor α
SHP: small heterodimer partner
SPMs: specialized pro-resolving mediators
TGFβ: transforming growth factor-β
TNFα: Tumor necrosis factor α
UGT: UDP-glucuronosyltransferase
UHPLC: Ultra-high-pressure liquid chromatography
URSO: ursodeoxycholic acid - Ursodiol®
