# Supplementary material for: Pharmaco-nutraceutical improvement of the response to obeticholic acid with omega-3 polyunsaturated fatty acids
Source: Biochem J. 2025 Aug 18;482(16):1145–61. doi: 10.1042/BCJ20253113 (PMC12493161; doi:10.1042/BCJ20253113)
Supplement: Online supplementary material 1 [file bcj-482-16-BCJ20253113-s001.docx]

**SUPPLEMENTAL MATERIALS**

**Supplementary Table 1: Primers and conditions used for quantitative real-time PCR experiments. Adapted from (**[**24**](#_ENREF_24)**,** [**25**](#_ENREF_25)**)**

| **Gene** | **Primers** | **Annealing Temperature**  **(°C)** | **RT dilution** |
| --- | --- | --- | --- |
| CYP27 | Sense: 5′-CGGCAACGGAGCTTAGAGG | 60°C | HepG2 1/200;  HH 1/500;  HepaRG 1/50 |
|  | Antisense: 5′-GGCATAGCCTTGAACGAACAG |  |  |
| CYP7A1 | Sense: 5′-AGAAGCATTGACCCGATGGAT | 59°C | HepG2 1/50;  HH 1/100;  HepaRG 1/50 |
|  | Antisense: 5′-AGCGGTCTTTGAGTTAGAGGA |  |  |
| CYP8B1 | Sense: 5′-GAAGCGCATGAGGACCAAG | 59°C | HepG2 1/50;  HH 1/500;  HepaRG 1/50 |
|  | Antisense: 5′-TTGCATATTGCCCAAAGTCTAGT |  |  |
| BSEP | Sense: 5′-GGGCCATTGTACGAGATCCTAA | 60°C | HH 1/100;  HepaRG 1/50; |
|  | Antisense: 5′-TGCACCGTCTTTTCACTTTCTG |  |  |
| MRP2 | Sense: 5′-CAAACTCTATCTTGCTAAGCAGG | 59°C | HepG2 1/1000;  HH 1/1000;  HepaRG 1/1000 |
|  | Antisense: 5′-TGAGTACAAGGGCCAGCTCTA |  |  |
| MRP3 | Sense: 5′-CAGAGAAGGTGCAGGTGACA | 59°C | HepG2 1/200;  HH 1/500;  HepaRG 1/50 |
|  | Antisense: 5′-CTAAAGCAGCATAGACGCCC |  |  |
| MRP4 | Sense: 5′-GGACAAAGACAACTGGTGTGCC | 64°C | HepG2 1/200;  HH 1/200;  HepaRG 1/50 |
|  | Antisense: 5′-AATGGTTAGCACGGTGCAGTGG |  |  |
| NTCP | Sense: 5′-TGATATCACTGGTCCTGGTTCTCA | 61°C | HepG2 1/50;  HH 1/500;  HepaRG 1/50 |
|  | Antisense: 5′-GCATGTATTGTGGCCGTTTG |  |  |
| BIP | Sense: 5′-CTTGGT ATTGAAACTGTGGGAGGTG | 60°C | HepG2 1/1000 |
|  | Antisense: 5′-TTCCAGTCAGATCAAATGTACCCAG |  |  |
| CHOP | Sense: 5′-GGAGGAGCCAGAACCAGCAGA | 60°C | HepG2 1/500 |
|  | Antisense: 5′-TTCCGTTTCCTGGTTCTCCCTT |  |  |
| COL1A1 | Sense: 5′-GTCGAGGGCCAAGACGAAG | 60°C | HepG2 1/50 |
|  | Antisense: 5′-CAGATCACGTCATCGCACAAC |  |  |
| TNFα | Sense: 5′-GAGGCCAAGCCCTGGTATG | 59°C | THP1 1/50 |
|  | Antisense: 5′-CGGGCCGATTGATCTCAGC |  |  |
| IL1β | Sense: 5′-TGAAGCTGATGGCCCTAAACA | 59°C | THP1 1/5000 |
|  | Antisense: 5′-GTAGTGGTGGTCGGAGATTCG |  |  |
| IL6 | Sense: 5′-CCTGACCCAACCACAAATGC | 59°C | THP1 1/500 |
|  | Antisense: 5′-CCTTAAAGCTGCGCAGAATGA |  |  |
| MCP1 | Sense: 5′-AGTGTCCCAAAGAAGCTGTGATC | 59°C | THP1 1/500 |
|  | Antisense: 5′-TCCAGGTGGTCCATGGAATC |  |  |

**Supplemental Table 2. Bile acids levels in media from human hepatoma HepG2 cells cultured in the presence of obeticholic acid, long-chain n-3 PUFAs, or both.**

|  | ***Bile acids levels*** | | | | ***p-values*** | | | |
| --- | --- | --- | --- | --- | --- | --- | --- | --- |
|  | ***Vehicle*** | ***OCA*** | ***EPA/DHA*** | ***OCA +***  ***EPA/DHA*** | ***OCA vs Vehicle*** | ***EPA/DHA***  ***vs Vehicle*** | ***OCA +***  ***EPA/DHA***  ***vs vehicle*** | ***OCA +***  ***EPA/DHA***  ***vs OCA*** |
| 7α-C4 | N.D | N.D | N.D | N.D | NA | NA | NA | NA |
| CA | N.D | N.D | N.D | N.D | NA | NA | NA | NA |
| CDCA | 8.3±1.0 | 4.3±0.1 | 4.3±1.1 | 2.6±0.3 | *0.0017* | *0.0026* | *0.0001* | *0.0314* |
| DCA | 0.2±0.3 | 0.0±0.0 | 0.3±0.3 | 0.3±0.2 | 0.4070 | 0.5367 | 0.8058 | 0.3901 |
| LCA | N.D | N.D | N.D | N.D | NA | NA | NA | NA |
| HDCA | N.D | N.D | N.D | N.D | NA | NA | NA | NA |
| HCA | N.D | N.D | N.D | N.D | NA | NA | NA | NA |
| UDCA | N.D | N.D | N.D | N.D | NA | NA | NA | NA |
| **Unconjugated bile acids** | **8.2±1.2** | **4.3±0.1** | **4.6±1.0** | **4.6±0.6** | ***0.0027*** | ***0.0035*** | ***0.0002*** | ***0.0083*** |
| GCA | 0.2±0.4 | 0.0±0.0 | 0.7±0.2 | 0.7±0.5 | 0.9909 | 0.0823 | 0.4669 | 0.4713 |
| GCDCA | 3.5±0.2 | 3.2±0.3 | 3.1±0.3 | 3.1±0.4 | 0.2258 | 0.1085 | 0.5996 | 0.5863 |
| GDCA | 1.1±0.1 | 1.2±0.2 | 1.1±0.1 | 1.1±0.3 | 0.7925 | 0.8430 | 0.5516 | 0.4675 |
| GLCA | N.D | N.D | N.D | N.D | NA | NA | NA | NA |
| GHDCA | N.D | N.D | N.D | N.D | NA | NA | NA | NA |
| GUDCA | N.D | N.D | N.D | N.D | NA | NA | NA | NA |
| TCA | 1.0±0.3 | 1.0±0.3 | 0.9±0.1 | 0.9±0.4 | 0.9961 | 0.62111 | 0.7221 | 0.3771 |
| TCDCA | 2.9±0.4 | 3.0±0.2 | 3.3±0.9 | 3.3±0.4 | 0.7655 | 0.4274 | 0.4717 | 0.3683 |
| TDCA | 1.2±0.3 | 1.0±0.1 | 1.1±0.2 | 1.1±0.5 | 0.4285 | 0.7294 | 0.4322 | 0.5135 |
| TLCA | N.D | N.D | N.D | N.D | NA | NA | NA | NA |
| THDCA | N.D | N.D | N.D | N.D | NA | NA | NA | NA |
| TUDCA | N.D | N.D | N.D | N.D | NA | NA | NA | NA |
| **Conjugated BAs** | **9.8±1.0** | **9.3±0.9** | **10.2±1.0** | **10.2±1.2** | **0.5396** | **0.6969** | **0.7479** | **0.3912** |
| **Primary BAs** | **15.5±1.9** | **11.5±0.8** | **12.2±1.4** | **12.2±0.7** | ***0.0184*** | ***0.0305*** | ***0.0026*** | **0.1937** |
| **Secondary BAs** | **2.5±0.5** | **2.2±0.2** | **2.5±0.4** | **2.5±0.8** | **0.4340** | **0.8753** | **0.6968** | **0.5929** |
| **TOTAL BAs** | **18.0±2.1** | **13.6±0.9** | **14.8±1.6** | **14.8±1.5** | ***0.0201*** | ***0.0488*** | ***0.0080*** | **0.5537** |

Human hepatoma HepG2 cells were treated with vehicle (DMSO and ethanol), obeticholic acid (OCA; 1μM) in the absence or presence of eicosapentaenoic and docosahexaenoic acids (EPA/DHA; 50/50μM) for 36H. Cell media were collected and profiled for the presence of 20 bile acids species using LC-MS/MS as detailed in the materials and methods section. Statistical significances as determined by a one-way ANOVA were:

- Vehicle vs OCA treated cells: *:*p*<0.05; **:*p*<0.01; ***:*p*<0.001.

- OCA *vs* OCA+EPA/DHA: §§:*p*<0.01; §§§:*p*<0.001.

7α-C4: 7α-hydroxy-4-cholesten-3-one; CA: cholic acid; CDCA: chenodeoxycholic acid; DCA: deoxycholic acid; LCA: lithocholic acid; HDCA: hyodeoxycholic acid; HCA: hyocholic acid; UDCA: Ursodeoxycholic acid; GCA: glycocholic acid; G: glyco-; T: tauro; N.D: Not detected; NA: not applicable.
